# Surgical removal of cesarean-related textiloma: A case report

**DOI:** 10.1016/j.ijscr.2025.111388

**Published:** 2025-04-28

**Authors:** Mohammad Al-Jawad, Nour Abdulazize Lbabidi, Mohammad Aldaher, Reema Khateeb, Aya Hamze, Aghyad Kurda Danial

**Affiliations:** University of Aleppo, Faculty of Medicine, Aleppo, Syria

**Keywords:** Textiloma, Gossypiboma, Cesarean section, Case report

## Abstract

**Introduction:**

Gossypiboma, a rare complication of surgery, involves retained surgical items like sponges, posing serious health risks and legal challenges. Though incidence rates have declined, the condition remains difficult to diagnose, often presenting with nonspecific symptoms and significant medicolegal implications.

**Case presentation:**

A 30-year-old female with persistent gastrointestinal symptoms post-cesarean section was diagnosed with a retained stitch after 1.5 years, leading to surgical intervention. Following the removal of the foreign body, her symptoms improved significantly, highlighting the importance of thorough evaluation in post-surgical patients.

**Discussion:**

Despite preventive measures, gossypibomas remain a significant issue, occurring in approximately 1 in 100 to 3000 surgical cases, particularly in emergency situations. Clinical manifestations can vary widely, and CT imaging is crucial for diagnosis, revealing characteristic patterns that guide surgical intervention for effective treatment.

**Conclusion:**

This case highlights the seriousness of gossypiboma and the need for increased medical awareness to prevent its occurrence. Early diagnosis and appropriate management are essential to avoid severe complications, emphasizing the importance of educating healthcare professionals about gossypibomas as a differential diagnosis.

## Highlights

• Gossypiboma remains a serious issue in surgery, necessitating increased medical awareness.
• Its symptoms can vary widely, making early diagnosis critical.
• This case underscores the importance of preventive measures to reduce risks associated with retained surgical materials.

## 1 Introduction

A gossypiboma, also known as a textiloma, is a rare but significant complication that can arise from any surgical procedure, posing serious health risks to patients and legal challenges for surgeons. Retained surgical items, particularly surgical sponges or gauzes, are the most frequently encountered issues due to their common usage and the difficulty in identifying them among surrounding tissue once they become saturated with blood. This complication not only endangers patient safety but also carries medicolegal implications for healthcare providers and institutions [1,2].

The intraperitoneal cavity is the most common site for gossypibomas. While the true incidence remains uncertain, there has been a positive trend indicated by a decline in detection rates over recent decades. Initially, the reported incidence was 1 in 1000 abdominal surgeries, but recent studies show it has decreased to between 0.08 and 0.18 per 1000. The identification rates of retained foreign bodies (RFB) vary by type of surgical procedure, with incidences of 17.69 % for cesarean sections, 16.33 % for abdominal hysterectomies, and 13.54 % for exploratory laparotomies in cases of acute abdomen [3].

The typical responses to retained surgical gauze include abscess formation accompanied by an exudative inflammatory reaction or the development of an aseptic mass with a fibrotic response. Patients may experience symptoms such as abdominal pain, nausea, vomiting, anorexia, and weight loss, which can result from obstructive or malabsorptive syndromes due to multiple intestinal fistulas or bacterial overgrowth within the lumen. Diagnosing gossypibomas is challenging, as they can resemble benign or malignant soft tissue tumors in the abdomen and pelvis, and their symptoms are often nonspecific, potentially emerging years after the surgical procedure [4].

In our case we present a case of A 30-year-old female diagnosed to have textiloma which cause the obstruction of the small intestine after cesarean section and she was treated successfully in our hospital, and we highlight the challenges encountered in managing this unique case as per the SCARE checklist [5].

## 2 Case presentation

### 2.1 History & initial symptoms

A 30-year-old female presented to our hospital with symptoms included nausea, diarrhea, vomiting, and abdominal pain, having undergone a cesarean section approximately 1.5 years prior.

One-week post-surgery, she experienced abdominal cramping, fever, and continued gastrointestinal symptoms, which led to a diagnosis of gastritis by her obstetrician, who prescribed appropriate treatment. Initially, her symptoms improved in the first month but then worsened, necessitating the use of painkillers. After three months, her condition deteriorated further, prompting an ultrasound that yielded normal results. Despite being prescribed medication that provided some relief, she continued to have follow-ups every two months, all of which showed normal examination findings. However, 1.5 years after the surgery, she began experiencing new symptoms, including difficulty passing gas, fever, chills, vomiting, and a general sense of malaise.

### 2.2 Diagnostic workup

Laboratory tests revealed several concerning values: a slightly elevated white blood cell count (WBC) of 10.49 × 10^9/L, indicating a possible immune response; low hemoglobin (HGB) at 10.93 g/dL and hematocrit (HCT) at 33.4 %, suggesting mild anemia; a mean corpuscular volume (MCV) at the lower end of normal at 80.2 fL, potentially indicating iron-deficiency anemia; and an elevated red cell distribution width (RDW-CV) of 16.0 %, reflecting variability in red blood cell size commonly associated with anemia.

**Fig. 1:**
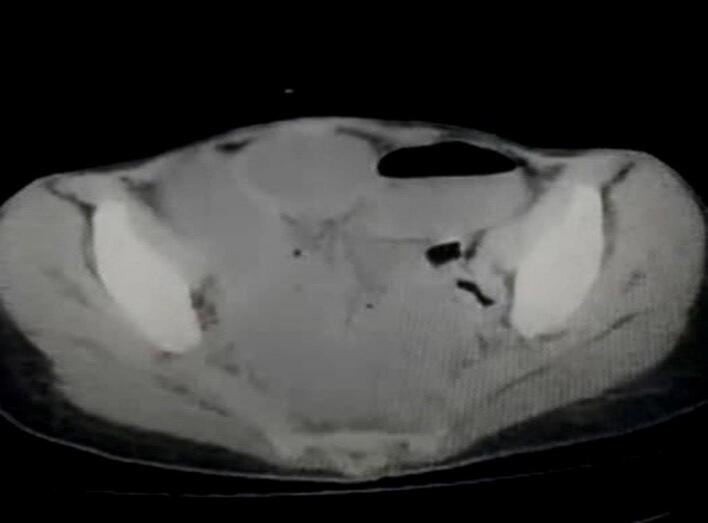
Axial CT scan demonstrates a radiopaque foreign body within the intestinal lumen, accompanied by mesenteric lymphadenopathy, ascites, and perilesional inflammatory stranding.

A CT scan revealed a foreign body within the intestinal tract, accompanied by mesenteric lymphadenopathy and ascites, as well as an inflammatory reaction surrounding the foreign body (Fig. 1).

### 2.3 Management & outcome

**Fig. 2:**
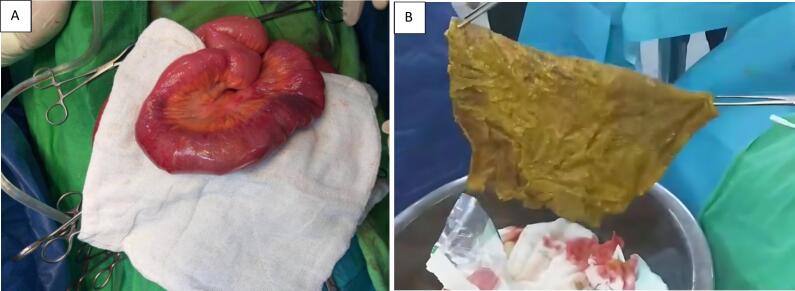
A) Intraoperative photograph depicts the extraction of a gossypiboma from the obstructed intestinal segment. B) Macroscopic examination reveals the retained surgical textile post-removal, exhibiting characteristic folded morphology.

Consequently, the patient underwent surgical intervention involving a midline incision to remove the identified migrated stitch. A suction drain was placed in the pelvic region, and saline lavage was performed to cleanse the area before closing the wound (Fig. 2(A, B)). Postoperatively, the patient was closely monitored for stabilization, and the intervention successfully alleviated her symptoms, leading to an improvement in her overall health before her discharge from the hospital.

## 3 Discussion

Despite the implementation of numerous preventive measures during surgical procedures, retained gauzes or sponges continue to pose a significant issue. Gossypiboma occurs in approximately 1 in every 100 to 3000 surgical cases and in 1 in every 1000 to 15,000 intra-abdominal surgeries. This complication is particularly prevalent in situations involving busy surgical environments, emergencies, unplanned procedural changes, and patients with a high body mass index [4].

The rise in incidence of gossypibomas has been linked to emergency surgical interventions, particularly in the obstetric field, such as cases of placenta previa, placenta accreta, hemorrhage, and uterine rupture. Emergency surgeries are identified as the most common risk factor for the occurrence of gossypibomas, accounting for 26 % of cases, closely followed by errors in sponge counting, which represent 25 % [6].

Several hypotheses have been put forward to explain the presumed transvisceral migration of foreign bodies such as surgical sponges. One hypothesis, derived from experimental studies, outlines four stages in this migration process. The first stage is the foreign body reaction, during which the sponge becomes encapsulated by the omentum and adjacent loops of intestine. This is followed by secondary infection, where cotton filaments penetrate the intestinal lumen, leading to cytolysis. The third stage involves the invasion of the sponge into the lumen, resulting in mass formation. Finally, the remodeling stage occurs, during which a fibrotic scar develops at the site of migration [7].

Clinical manifestations of gossypibomas can vary significantly, ranging from asymptomatic cases that are discovered incidentally to severe complications, including bowel perforation, obstruction, fistula formation, or sepsis [8].

In our case, the patient exhibited several distinctive symptoms, including nausea, diarrhea, vomiting, and abdominal pain. Additionally, she experienced abdominal cramping and fever. As her condition progressed, she developed difficulty passing gas, chills, and a general sense of malaise. These symptoms collectively highlight the complexities associated with gossypibomas.

CT imaging is the preferred method for detecting gossypibomas and their complications. Gossypibomas are typically identified by a low-density heterogeneous mass with a high-density external wall, exhibiting a spongiform pattern with air bubbles, which becomes more distinct with contrast enhancement. A radiopaque marker strip may appear as a thin metallic density within the mass, and calcifications can be observed on the mass wall. One case reported a long-standing gossypiboma as a cystic lesion with a thick calcified rind, introducing the calcified reticulate rind sign. The spongiform pattern with gas bubbles is the most characteristic CT sign of gossypibomas [9].

In our case, a CT scan revealed a foreign body within the intestinal tract, along with mesenteric lymphadenopathy and ascites. Additionally, there was an inflammatory reaction surrounding the foreign body, which aligned with the typical findings associated with gossypibomas. This combination of imaging results was crucial in confirming the diagnosis and guiding the subsequent surgical intervention.

Surgical intervention is the primary treatment for gossypiboma, highlighting the necessity of early detection and prompt removal to reduce the risk of complications. Prevention strategies, supported by organizations like the American College of Surgeons (ACS) and the Association of Registered Nurses of the USA, stress the importance of systematic counting procedures at various stages of surgery. This includes counts before, during, and after procedures, with mandatory exploration of the surgical site in cases of any discrepancies [10].

In our case, the patient received prompt surgical intervention through a midline incision to excise the migrated stitch. To ensure optimal recovery, a suction drain was strategically placed in the pelvic region, followed by saline lavage to thoroughly cleanse the area before wound closure. The postoperative phase involved meticulous monitoring to ensure the patient's stabilization. This decisive intervention not only alleviated her symptoms but also significantly enhanced her overall health, allowing for a successful discharge from the hospital. This case exemplifies the vital importance of timely surgical action in the management of gossypibomas.

To prevent retained surgical items post-cesarean, strict adherence to double-count protocols for sponges/instruments is essential. Radiopaque-tagged materials and emerging technologies like RFID tracking enhance detection. Mandatory intraoperative imaging for high-risk cases and staff training on documentation standards further reduce risks. Early inclusion in differential diagnoses for unexplained abdominal symptoms can mitigate complications.

## 4 Conclusion

This case highlights the significant risks posed by gossypiboma and demonstrates the necessity for increased medical vigilance to prevent such occurrences. Retained surgical materials may cause severe complications if not detected and managed promptly. Early and accurate diagnosis plays a crucial role in ensuring proper treatment and preventing misdiagnosis that could lead to ineffective interventions. Raising awareness among healthcare providers about considering gossypiboma in differential diagnoses improves patient outcomes and reduces risks associated with this preventable condition.

## CRediT authorship contribution statement

The work's conception and design: Mohammad Al-Jawad
Paper writing, and article revision: Mohammad Al-Jawad, Mohammad Aldaher, Reema Khateeb, Aya Hamze
Final revision and approval: Nour Abdulazize Lbabidi, Aghyad Kurda Danial.

## Informed consent

Informed consent was obtained from the patient for publication of this case report details.

## Consent for publication

All authors provide consent for publication.

## Ethical approval

Not applicable.

## Guarantor

Aghyad Kurda Danial.

## Provenance and peer review

Not commissioned, externally peer-reviewed.

## Funding

There are no funding sources.

## Declaration of competing interest

The authors declare that they have no competing interests.
